# Phase-Synchronized Learning of Periodic Compliant Movement Primitives (P-CMPs)

**DOI:** 10.3389/fnbot.2020.599889

**Published:** 2020-11-12

**Authors:** Tadej Petrič

**Affiliations:** CoBoTaT Lab, Department of Automatics, Biocybernetics and Robotics, Jožef Stean Institute (JSI), Ljubljana, Slovenia

**Keywords:** adaptive control, autonomous learning, human-robot interaction, internal dynamic model, compliant movement primitives

## Abstract

Autonomous trajectory and torque profile synthesis through modulation and generalization require a database of motion with accompanying dynamics, which is typically difficult and time-consuming to obtain. Inspired by adaptive control strategies, this paper presents a novel method for learning and synthesizing Periodic Compliant Movement Primitives (P-CMPs). P-CMPs combine periodic trajectories encoded as Periodic Dynamic Movement Primitives (P-DMPs) with accompanying task-specific Periodic Torque Primitives (P-TPs). The state-of-the-art approach requires to learn TPs for each variation of the task, e.g., modulation of frequency. Comparatively, in this paper, we propose a novel P-TPs framework, which is both frequency and phase-dependent. Thereby, the executed P-CMPs can be easily modulated, and consequently, the learning rate can be improved. Moreover, both the kinematic and the dynamic profiles are parameterized, thus enabling the representation of skills using corresponding parameters. The proposed framework was evaluated on two robot systems, i.e., Kuka LWR-4 and Franka Emika Panda. The evaluation of the proposed approach on a Kuka LWR-4 robot performing a swinging motion and on Franka Emika Panda performing an exercise for elbow rehabilitation shows fast P-CTPs acquisition and accurate and compliant motion in real-world scenarios.

## 1. Introduction

Programming by demonstration (PbD) is a typical approach for transferring skills to robots by mirroring human actions (Billard et al., 2008; Argall et al., 2009; Calinon et al., 2014; Peternel et al., 2018). For simple tasks, human demonstrations are typically recorded using vision-based systems (Welschehold et al., 2016) or motion tracking suites (Filippeschi et al., 2017). For a more challenging task where force constraints and compliance strategies are required, the kinesthetic guidance or multi-modal human-in-the-loop skill transfer approaches can be used (Peternel et al., 2014; Rozo et al., 2016). Besides, such learning has the advantage of already being adapted to the kinematic and dynamic parameters of the robotic system. Here, typically a well-established inverse dynamic control approach is in use (Sciavicco and Siciliano, 2012). However, due to the increasing complexity of robot mechanisms and tasks, they are performing, accurate dynamical models' of both the robot and the task are often difficult to obtain. To bridge the gap, machine learning algorithms were adopted because of their ability of learning complex models. Although learning algorithms are powerful enough to learn the inverse dynamics of both the robot and the task (Nguyen-Tuong and Peters, 2011), they still require a large amount of data for the learning processes, which makes them unsuitable for on-line learning of tasks-specific dynamics.

Knowing the exact dynamical model is crucial to achieving compliant robot behavior, which is needed when robots are operating in an unstructured environment. Hence, exact dynamical models of both, the robot and the task makes it possible to either adjust the controller feedback gains to obtain the desirable compliance or to prescribe the desired dynamic behavior (Buchli et al., 2011; Kronander and Billard, 2013; Žlajpah and Petrič, 2019). Skill learning approaches that can expand the database can be time-consuming. For example, reinforcement learning might take a long time to tune the skill because a high number of repetitions is needed (Kober et al., 2012). Such exploitative learning methods were successfully used for learning force profiles for in-contact tasks (Racca et al., 2016). On the other hand, supervised learning methods are typically faster but require a reference for the optimization process (Wang et al., 2009). Nevertheless, even these methods might take too much time to produce a large enough database enabling statistical methods to generate an accurate dynamic model for a given task. However, when using PbD methods, dynamic models of both the robot and the task, are usually not known and can not be easily learned from imitation. Since modeling of system dynamics is typically a difficult and time-consuming task, this work instead addresses the problem of how to obtain the task-specific dynamics through autonomous learning and thereby avoid the need for an expert to define them.

Learning of task-specific dynamics was proposed in Deniša et al. (2016), where Compliant Movement Primitives (CMPs) were introduced. CMPs encode both the kinematic trajectory in the form of Dynamic Movement Primitives (DMP) (Ijspeert et al., 2013) and accompanying dynamics called Torque Primitives (TPs), i.e., joint-torques encoded with weighted radial-basis functions. In Deniša et al. (2016) TPs were obtained through execution of the desired movement trajectories using high-gain feedback control. This limitation was mitigated in Petrič et al. (2018), where TPs were learned iteratively until the error of compliant tracking was reduced below a predefined threshold.

The main contribution of this paper is a two-layered system that combines Phase-synchronized Adaptive Fourier Series (P-AFO) with Periodic Compliant Movement Primitives (P-CMPs). The P-AFO is an incremental improvement of AFO proposed in Petrič et al. (2011), which guaranties unambiguous frequency and phase synchronization to an arbitrary input signal, which is crucial for P-CMPs. Furthermore, the P-CMPs is a periodic extension of CMPs proposed in Deniša et al. (2016) and Petrič et al. (2018). Here the kinematic trajectory is encoded in Periodic Dynamic Movement Primitives (Gams et al., 2009) and the corresponding task-specific dynamics with Periodic Torque Primitives (P-TPs). For the P-TPs we propose a novel combination of weighted kernel functions that are frequency and phase-dependent. The novel P-TPs framework allows direct modulation of frequency, which was not possible before (Deniša et al., 2016). Inspired by human sensorimotor learning (Kawato, 1990), the P-TPs are learned on-line using a feed-back error learning approach. The learning is active until the tracking error of a compliant controller robot is reduced below a predefined threshold.

This paper is organized as follows. In the next section, we describe related work detailing the topics of learning of robot torque profiles and their modulation and generalization. In section 3 we describe the main contributions of this paper, i.e., unambiguous phase synchronization (P-AFO), periodic torque primitives (P-TPs), and the integration of feedback error learning. Results of experimental evaluation on a Kuka LWR-4 robot arm learning to perform a swinging task and evaluation on Franka Emika Panda robot learning to rehabilitate the elbow by a stretching task are presented in section 4. A discussion concludes the paper in section 5.

## 2. Related Work

### 2.1. Torque Learning

For accurate and compliant execution of tasks, the task-space dynamics is required (Del Prete and Mansard, 2016), whereby a dynamic model of the task might be hard to obtain (Petrič et al., 2010). To mitigate mathematical modeling, different biologically inspired methods were proposed to enhance robot control (Franklin and Wolpert, 2011). Merging them with robots that have joint-torque sensors led to the development of Compliant movement Primitives (CMPs), first reported in Petrič et al. (2014). Originally, CMPs recorded feed-forward torques during initial task execution with stiff robot behavior that ensures accurate motion tracking. Once torques were recorded, they were used as feed-forward components of the CMPs in the next repetitions of the same task. Since the torque profiles had to be recorded for each variation of the tasks, even for different execution speeds, a statistical generalization method was proposed in Deniša et al. (2016). They showed that generalization between CMPs can successfully be used to generate CMPs for tasks where kinematic or dynamic parameters were changed. Besides generalization, a statistical-graph search was shown to effectively generate new CMPs by joining together different parts of several CMPs (Deniša et al., 2013).

Exploiting the feed-forward torque was also utilized when the possibility of measuring joint torque was available. For example in Calandra et al. (2015) they use tactile sensors to compute joint torques on an iCub humanoid robot. The computed joint-torques were used as feed-forward signals similar to the CMPs framework. Learning of joint torques together with kinematic trajectory was also implemented in Steinmetz et al. (2015), where the recorded torques were used as a feed-forward signal to increase the motion accuracy of the in-contact task. Originally the learning of CMPs torque signals was performed during an exact motion execution, whereby the robot was stiff due to the high feedback gains. As a consequence, the application of CMPs during learning was limited and potentially dangerous when interacting with the environment or humans. To mitigate this issue, an approach using autonomously learning of torque profiles while using compliant robot behavior, i.e., low feed-back gains, was introduced in Petrič et al. (2018). However, the approach was not suitable for periodic tasks.

Other approaches for torque learning not directly related to CMPs were also proposed. Gaussian process regression for on-line learning of the dynamical model was proposed in Nguyen-Tuong and Peters (2011), where the accuracy of the dynamical model was improved while keeping compliant robot behavior. While results were promising, this approach required a large amount of data, hence it was not focused on learning only task-specific torques. For learning only task-specific torques iterative learning control (ILC) was utilized in Schwarz and Behnke (2013). Here ILC was used to identify model parameters for motor and friction models. Similarly, in Kronander et al. (2015) ILC was used to update the dynamical model. Inspired by human sensorimotor learning, Kawato (1990) introduced a feedback error learning approach for learning task-specific dynamics for a given kinematic trajectory. The feedback error learning was later adopted in Gopalan et al. (2013), where it was used to stabilize the controller's output for adapting the gait of an under-actuated bipedal robotic system.

### 2.2. Modulation and Generalization

Trajectory modulation and generalization is a wide topic that can be considered from different domains of application. Mostly, methods for modulation and generalization were focused on the kinematic trajectory and only a few dealt with dynamics. The modulation and generalization ability of kinematic and dynamic parameters are specifically important for the P-CMPs framework proposed in this paper. The kinematic part of P-CMPs is encoded with P-DMPs, which already allow a certain degree of modulation and generalization. In Gams et al. (2009), the modulation abilities of DMPs to change the goal and the frequency was demonstrated. Furthermore, the DMPs were also used as means to represent results of statistical generalization using locally weighted regression in Ude et al. (2010) and generalization between weights of DMPs using Gaussian process regression (GPR) in Forte et al. (2012). For both approaches, a task parameter is required to generate a new trajectory from a motion database. Similarly, in Stulp et al. (2013) the task parameter was used to learn the weights of DMPs of a single demonstration. Instead of rallying on one demonstration, Matsubara et al. (2011) used several demonstrations to create a parametric attractor landscape in a set of differential equations. Similarly, a variation of DMPs as a Mixture of Motor Primitives (MoMP) was introduced in Mülling et al. (2013), where they proposed an algorithm that can autonomously update the weights. By exploiting the external inputs the on-line modulation and adaptation of DMPs are also possible as shown in Gams et al. (2010) and Kulvicius et al. (2013).

The DMPs are not the only trajectory representation method or even the only dynamical systems used for modulation and generalization. However, because our proposed approach in this paper is composed also of DMPs, other possible alternatives are only briefly listed below. The task-specific Gaussian Mixture Models (TP-GMM) were proposed by Khansari-Zadeh and Billard (2011) and were also used in Calinon (2016). Another possibility is also Hidden Markov Models (HMM) that were used in Lee and Ott (2011). While these approaches rely on generating trajectories based on existing database entries, the trajectory generation based on extrapolation and the database expansion is still an open research topic. Extrapolation was mentioned in Calinon et al. (2013), where statistical methods were used to encode the movements. The algorithm for autonomous database expansion was proposed in Petrič et al. (2018), where the new compliant motion trajectories were generated also by extrapolating the database. The literature related to modulation and generalization of dynamic parameters is even more sparse. Besides already mentioned (Calinon et al., 2013; Deniša et al., 2013; Petrič et al., 2018), modulation, and generalization of dynamic parameters, such as forces and torques, was also researched in Gams et al. (2015), where a statistical generalization was used on force-based coupling terms. However, their approach was limited, since it requires user interaction to generate new database entries. Similarly, in Koropouli et al. (2015) a new policy was proposed where the input was motion data and the output was a force.

The generalization of both kinematic trajectories and torque profiles has been reported with the aforementioned CMPs in Deniša et al. (2013) and later extend with an approach enabling autonomous learning in Petrič et al. (2018). Our paper extends the approach in Deniša et al. (2013) and Petrič et al. (2010) first by introducing the Periodic-CMPs framework and second by proposing novel P-TPs formulation which includes frequency modulation capabilities.

## 3. Periodic Compliant Movement Primitives

**Figure 1:**
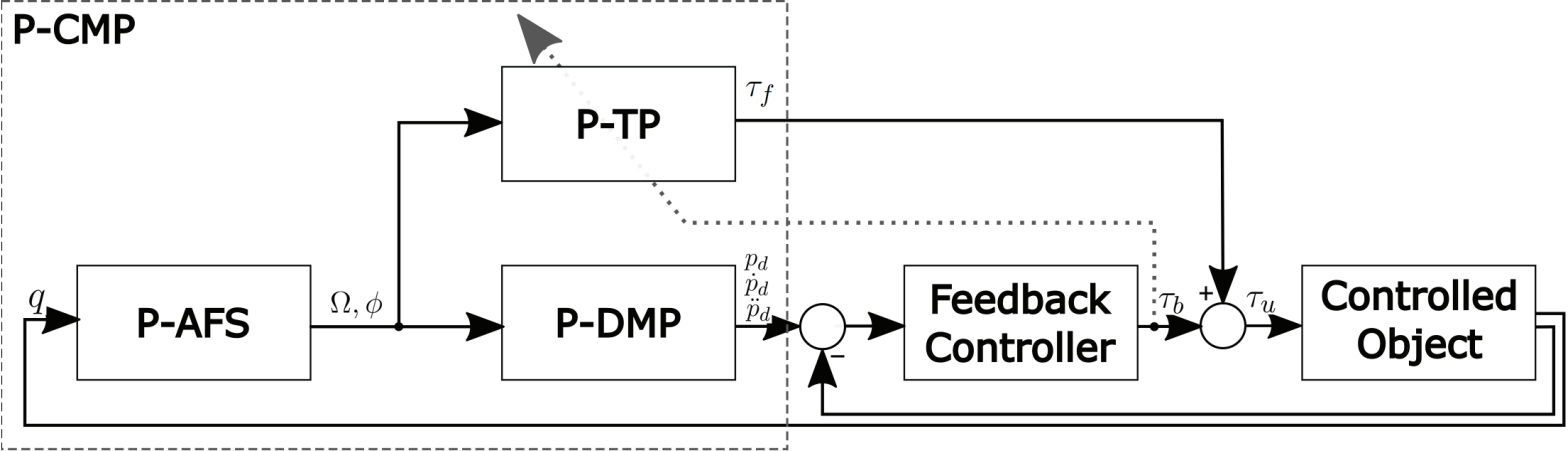
The multi-layered structure of the control system based on P-CMPs. The input *q* is a measured quantity and the output is the desired kinematic trajectory $p_{d};{ṗ}_{d};{\ddot{p}}_{d}$ and the corresponding torque profiles τ_*f*_. Note that the system can work in parallel for an arbitrary number of dimensions.

The inspiration for the P-CMPs multi-layered framework has been taken from the two-layered imitation system reported in Gams et al. (2009) and Petrič et al. (2011). In their work, the authors introduced a system that can be used for imitation learning, because it allows autonomous frequency adaption and learning of kinematic trajectories. The extension of kinematic trajectory with corresponding dynamic parameters in the form of P-CMPs is proposed in this paper and illustrated in Figure 1.

Periodic Compliant Movement Primitives (P-CMPs) *h*(Ω, ϕ) are defined as a combination of the adaptive oscillators (P-AFO), kinematic trajectories encoded in periodic Dynamic Movement Primitives (P-DMPs) and corresponding task-specific dynamics encoded in Periodic Torque Primitives (P-TPs).

(1) $$\begin{array}{l} h\left(\Omega ,\phi \right)=\left[p_{d}\left(\Omega ,\phi \right);{ṗ}_{d}\left(\Omega ,\phi \right);{\ddot{p}}_{d}\left(\Omega ,\phi \right);\tau_{f}\left(\Omega ,\phi \right)\right]. \end{array}$$

Here Ω and ϕ are the desired motion frequency and phase, respectively. ${\ddot{p}}_{d}\left(\Omega ,\phi \right)$, *p*_*d*_(Ω, ϕ) and ṗ_*d*_(Ω, ϕ) are the desired acceleration, velocity and position trajectories, respectively, encoded within P-DMPs. τ_*f*_(Ω, ϕ) are the corresponding joint torques encoded in P-TPs.

Similar to the discrete CMPs, the two-stage process is used to obtain the P-CMPs. First, the kinematic motion trajectories are obtained typically by imitation learning (Gams et al., 2009). Next, the corresponding periodic torques are obtained using the feedback error learning approach (Kawato, 1990).

### 3.1. Phase-Synchronization

The adaptive phase oscillator with the adaptive Fourier series was originally proposed in Petrič et al. (2011). The core is a second-order system of differential equations governed by

(2) $$\begin{array}{l} \dot{\phi }=\Omega -\kappa \epsilon \sin\left(\phi \right), \end{array}$$

(3) $$\begin{array}{l} \dot{\Omega }=-\kappa \epsilon \sin\left(\phi \right). \end{array}$$

Here Ω is the estimated motion frequency, κ is the coupling strength, ϕ is the corresponding phase and ϵ is governed by

(4) $$\begin{array}{l} \epsilon =q-\hat{q}, \end{array}$$

where *q* is the input signal and $\hat{q}$ is the estimated oscillator feedback. It is governed by

(5) $$\begin{array}{l} \hat{q}=\overset{m}{\underset{i=0}{\sum }}A_{i}\cos\left(i\phi \right)+\overset{m}{\underset{j=2}{\sum }}B_{j}\sin\left(j\phi \right) \end{array}$$

where *m* denotes the size of the modified Fourier series. Note that in this paper the sinus part of the series starts with index *j* = 2, which ensures unambiguously defined phase synchronization. The parameters *A*_*i*_ and *B*_*j*_ are updated as in Petrič et al. (2011).

(6) $$\begin{array}{l} {Ȧ}_{i}=\eta \epsilon \cos\left(i\phi \right),\;i\in \left[0,1,...,m\right], \end{array}$$

(7) $$\begin{array}{l} {Ḃ}_{j}=\eta \epsilon \sin\left(j\phi \right),\;j\in \left[2,3,...,m\right], \end{array}$$

where η is the parameter update rate. By skipping the first parameter of the sinusoidal part of the Fourier series, i.e., *j* = 1, we ensure the phase is always well-defined with respect to the input signal *q*. This is because the main frequency component and corresponding phase is now only related to cosine and not from a combination of cosine and sine as it was in the original system. The novel system is denoted with Phase-synchronized Adaptive Fourier Series (P-AFO). The evaluation results, focused on the novelty, i.e., phase synchronization, are shown in section 4.1.

### 3.2. Motion Trajectories

The second layer ensures the proper waveform of the kinematic trajectories. It is encoded by P-DMPS, which are anchored to the phase signal ϕ of the adaptive oscillator as in Petrič et al. (2011). The equations for a single-degree-of-freedom are summarized from Ijspeert et al. (2013). The second-order dynamic system of P-DMP is governed by

(8) $$\begin{array}{l} ż=\Omega \left(\alpha_{z}\left(\beta_{z}\left(g-y\right)-z\right)+f\right), \end{array}$$

(9) $$\begin{array}{l} ẏ=\Omega z, \end{array}$$

where α_*z*_ and β_*z*_ are the positive constants, which guarantee that the system monotonically converges, *g* is the center of oscillation, and *f* is the non-linear forcing term that determines the shape of the trajectory. It is given by

(10) $$\begin{array}{l} f\left(\phi \right)=\frac{\overset{N}{\underset{i=1}{\sum }}w_{i}\psi_{i}\left(\phi \right)}{\overset{N}{\underset{i=1}{\sum }}\psi_{i}\left(\phi \right)}. \end{array}$$

Here *w* is the vector determining the shape and ψ are the Gaussian-like kernel functions given by

(11) $$\begin{array}{l} \psi_{i}\left(\phi \right)=\exp\left(h\left(\cos\left(\phi -c_{i}\right)-1\right)\right), \end{array}$$

where *N* is the number of kernels, *h* are the kernels width and *c*_*i*_ is their distribution concerning the phase. Typically they are spread equally between 0 and 2π.

To learn the shape of the trajectory different methods where proposed. When data is available upfront, a batch regression can be used as in Ude et al. (2010). Alternatively, when learning on-line, recursive locally weighted regression can be used. The equations summarizing online learning for the incremental learning approach are summarized from Gams et al. (2009). By rewriting Equations (8) and (9) as one second-order differential equation we get

(12) $$\begin{array}{l} f_{d}=\frac{{\ddot{y}}_{d}}{\Omega^{2}}-\alpha_{z}\left(\beta_{z}\left(g-y_{d}\right)-\frac{{ẏ}_{d}}{\Omega }\right). \end{array}$$

Here the triplet of *y*_*d*_, ẏ_*d*_ and ${\ddot{p}}_{d}$ denotes the desired position, the velocity and the acceleration. To update the weights *w*_*i*_ of the kernel function ψ_*i*_, we use the flowing recursive least-squares method.

(13) $$\begin{array}{l} P_{i}\left(t+1\right)=\frac{1}{\lambda }\left(P_{i}\left(t\right)-\frac{P_{i}{\left(t\right)}^{2}}{\frac{\lambda }{\psi_{i}\left(\phi \left(t\right)\right)}+P_{i}\left(t\right)}\right), \end{array}$$

(14) $$\begin{array}{l} w_{i}\left(t+1\right)=w_{i}\left(t\right)+\psi_{i}\left(\phi \left(t\right)\right)P_{i}\left(t+1\right)e_{r}\left(t\right), \end{array}$$

(15) $$\begin{array}{l} e_{r}\left(t\right)=f_{d}\left(t\right)-w_{i}\left(t\right). \end{array}$$

The regression typically starts with *w*_*i*_ = 0 and *P*_*i*_ = 0. Note that *P*_*i*_ is the inverse covariance. λ is the forgetting factor.

Essentially the combination of P-AFO and P-DMP ensures robustness against perturbations and allows frequency modulation of the trajectory. Especially frequency modulation is crucial when performing human-robot cooperative tasks.

### 3.3. Torque Trajectories

The third layer encodes the corresponding torque trajectories τ_*f*_(Ω, ϕ) and it is denoted by P-TPs. Note that torques are task-specific, which means they are dependent on the dynamic properties of the task including the execution speed, e.g., frequency. Therefore we propose that P-TPs τ_*f*_(Ω, ϕ) are both, phase ϕ and frequency Ω dependent. They are governed by

(16) $$\begin{array}{l} \tau_{f}\left(\Omega ,\phi \right)=\frac{\overset{M}{\underset{i=1}{\sum }}\overset{K}{\underset{j=1}{\sum }}\nu_{i,j}\psi_{i}\left(\phi \right){\text{Ψ}}_{j}\left(\Omega \right)}{\overset{M}{\underset{i=1}{\sum }}\overset{K}{\underset{j=1}{\sum }}\psi_{i}\left(\phi \right){\text{Ψ}}_{j}\left(\Omega \right)} \end{array}$$

where ν is a *M* × *K* matrix that encodes the torque profiles and ψ and Ψ are the Gaussian like kernel functions given by

(17) $$\begin{array}{l} \psi_{i}\left(\phi \right)=\exp\left(h^{\phi }\left(\cos\left(\phi -c_{i}^{\phi }\right)-1\right)\right), \end{array}$$

(18) $$\Psi_{j}(\Omega )=\exp(-h^{\Omega }{\left(\Omega -c_{j}^{\Omega }\right)}^{2}).$$

Here, *h*^ϕ^ are the width of the kernel and $c_{i}^{\phi }$ is their distribution concerning the phase spread equally between 0 and 2π. *h*^Ω^ are the kernels width and $c_{j}^{\text{Ω}}$ is their distribution concerning the frequency. Typically $c_{j}^{\text{Ω}}$ is equal between 0 and 4π. Note that *M* is the number of phase kernels, and *K* is the number of frequency kernels.

The P-TPs are learned on-line while executing the encoded DMP motion with low gain impedance control using the following law

(19) $$\begin{array}{l} \tau_{u}=\tau_{b}+\tau_{f}, \end{array}$$

(20) $$\begin{array}{l} \tau_{b}=K_{p}e+K_{d}ė+K_{i}ë \end{array}$$

Here, *e*, ė, and ë are the differences between desired *p*_*d*_, ṗ_*d*_, and ${\ddot{p}}_{d}$ and actual position *p*, velocity ṗ, and acceleration $\ddot{p}$, respectively. *K*_*p*_, *K*_*d*_, and *K*_*i*_ are the constants selected to ensure robot behaves compliantly, i.e., set to match the low impedance control requirements.

To learn task-specific torque profiles, we used the feedback error learning approach (Nakanishi and Schaal, 2004). It is governed by

(21) $$\begin{array}{l} {\dot{\nu }}_{i,j}=\iota \tau_{b}, \end{array}$$

where ι is a positive constant determining the rate of learning. Note that stability analysis was given in Nakanishi and Schaal (2004).

Because the torques are updated on-line, the task performance, i.e., tracking accuracy, improves over time even if the feedback gains are low. The main idea used in the proposed P-CMPs framework approach is to assure the nominal behavior of the robot for the given periodic task even if compliant robot control is used, i.e., using low feedback gains. In this way, we can assure both, the good tracking accuracy and the compliant behavior. This increases safety aspects for robots working in an unstructured environment or with humans.

## 4. Experimental Validation

In this section we describe the simulations used to compare the P-AFO phase and frequency synchronization performance with the original AFO (Petrič et al., 2011); and two examples of P-CMPs applications with real-world robots, i.e., Kuka LWR-4 and Franka Emika Panda. Note that stability proofs of CMPs system and the AFO systems were already shown in the above-mentioned research (Nakanishi and Schaal, 2004; Petrič et al., 2011, 2018; Deniša et al., 2016). We therefore focused the evaluation on the system improvements and innovations.

### 4.1. P-AFO Evaluation

In this numerical simulation example, we compare the phase and frequency synchronization abilities of the original AFO system with the proposed P-AFO system. Note that in both cases the adaptation is done without any signal processing since the entire process of frequency and phase synchronization is completely embedded in the dynamics of the oscillator. In the following example we used for both, AFO and P-AFO, the flowing parameters: κ = 20, μ = 2, *m* = 4, and *A*_*i*_(0) = *B*_*j*_(0) = 0.5. The input *q* was a sinusoidal signal with a frequency of 1 Hz.

**Figure 2:**
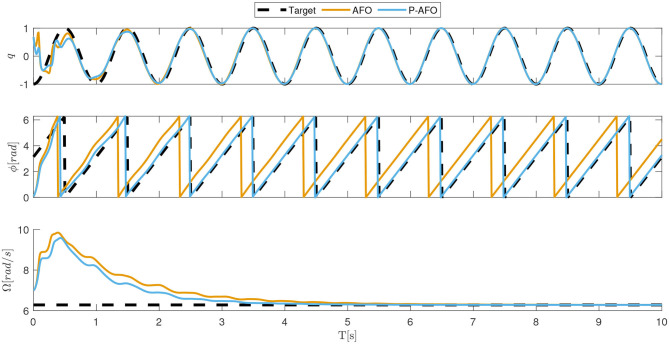
Typical convergence of an AFO and P-AFO systems driven by a sinusoidal periodic signal. In the top plot, the comparison between the input signal and the approximation of the system is shown. The middle plot shows the phase synchronization and the bottom plot shows the frequency adaption.

Frequency and phase adaption results are illustrated in Figure 2. We can see in the top plot that there is no difference between AFO and P-AFO systems performance regarding the adaptation toward the input signal. Similarly, we can also see in the bottom plot that there is no difference between AFO and P-AFO in frequency extraction performance. This shows and confirms that both systems can correctly adapt to extract the frequency of the input signal. However, the crucial difference is in the ability to unambiguously extract the phase signal from the input signal. Clearly, the original AFO signal can extract the phase, which is synchronized to the input signal. However, due to the sum of the first sinusoidal and cosinusoidal elements in the adaptive Fourier series in the original AFO system, a phase shift between the input signal and the extracted phase might appear.

**Figure 3:**
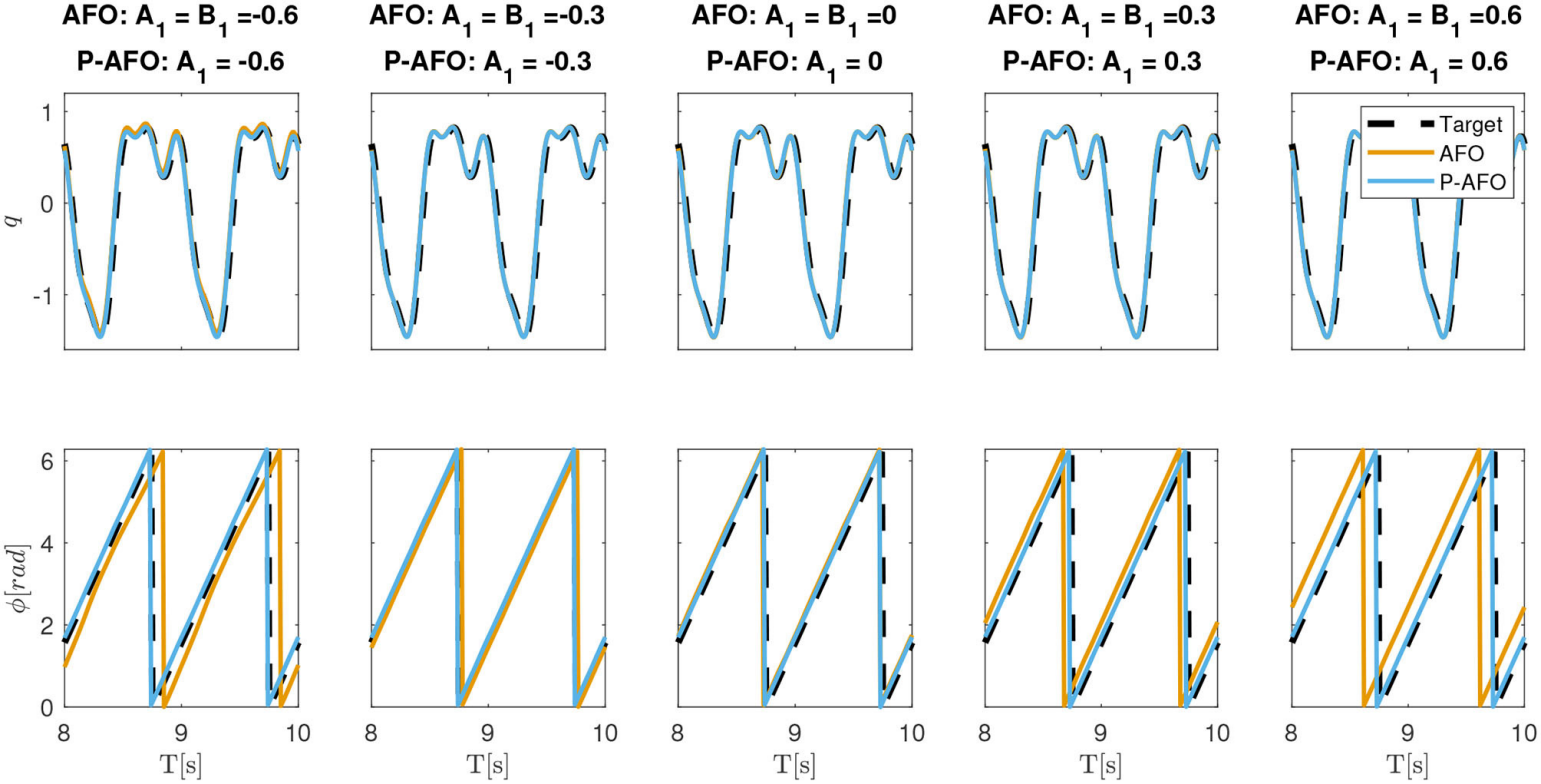
Typical convergence of an AFO and P-AFO systems driven by a periodic signal with different initial conditions. Top plots, shows the comparison between the input signal and the approximation of the system and middle plots shows the phase synchronization.

Figure 3 shows the adaptation to the periodic signal with different initial conditions of parameters *A*_*i*_ and *B*_*j*_. The results shows that the phase synchronization of the original AFO concerning the input signal is not repeatable. Note, that if we change the initial parameters or the start of the input signal, the phase shift between the input signal and the extracted phase of AFO will be different. Extracting the exact phase of the input signal is crucial for the P-CMPs. In the middle plot of Figure 2 and on the bottom plot of Figure 3, we can see that the proposed P-AFO system ensures that the phase is always unambiguously defined concerning the input signal. This allows us to precisely anchor the P-TPs to the corresponding P-DMPs, which, therefore, provides all the aforementioned advantages of a P-CMPs system.

### 4.2. Robot Dynamics Learning Example

**Figure 4:**
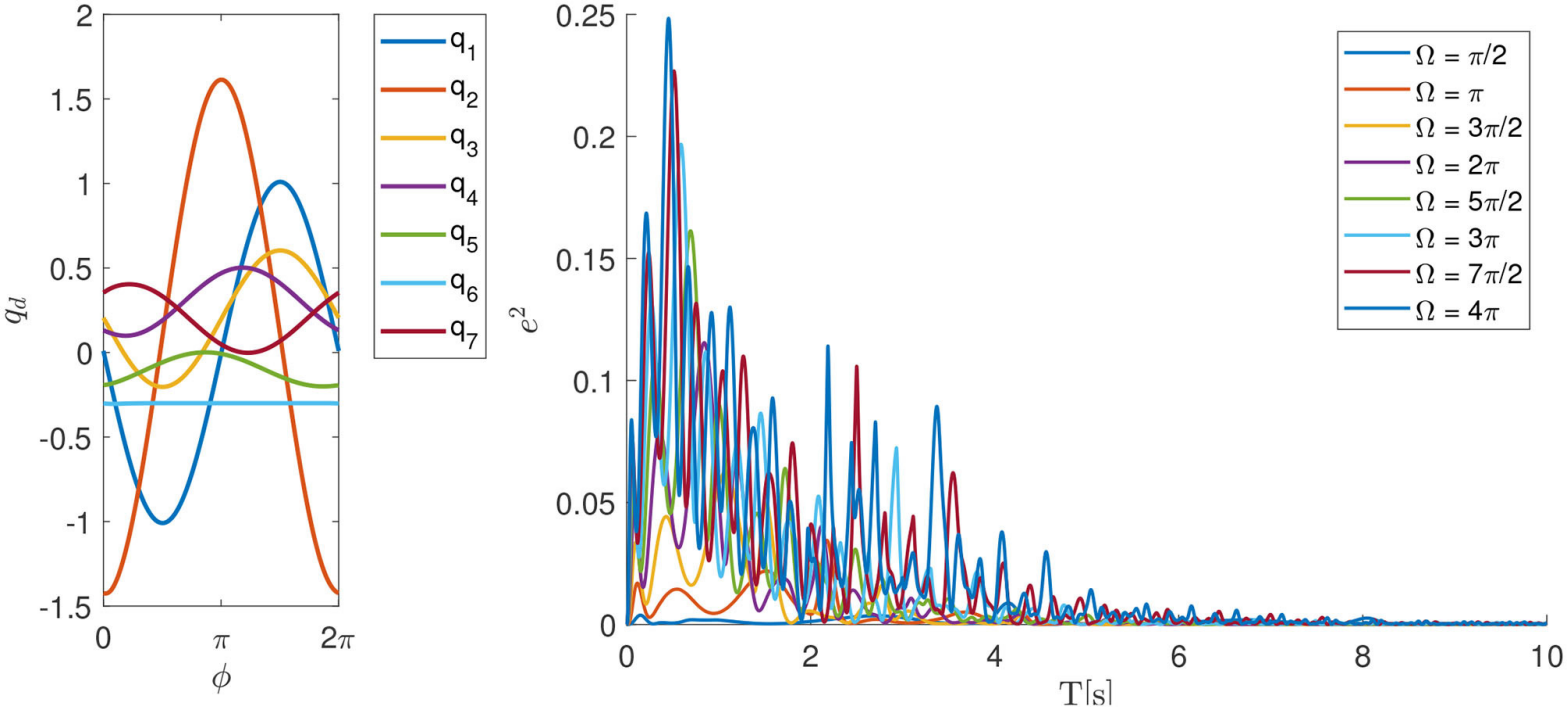
Learning of internal dynamical models for different motion frequencies on 7 degrees of freedom Kuka LWR-4 robot. The left plot shows the desired kinematic motion *q*_*d*_ dependent on the phase parameter ϕ and the right plot shows the sum of square motion tracking error during the leaning process.

To illustrate the ability to learn the internal dynamical model, we implemented the P-CMPs approach on a real robot Kuka LWR-4. In this example, the goal was to learn the corresponding dynamical model in P-TPs using the approach proposed in section 3. The kinematic trajectory for this task was predefined for all 7 degrees of freedom and it is shown on the left hand side of Figure 4. The robot feedback loop gains for all joints were set to 50 Nm/rad and the feed-back error learning parameter ι was set to 10. Note that in general the dynamical model of the robot is not strictly necessary for the proposed approach, however, we made use of the dynamical model provided by the Kuka controller. Even so, by using the provided dynamical model the tracking accuracy is still poor with selected feed-back gain as shown in Deniša et al. (2016).

**Figure 5:**
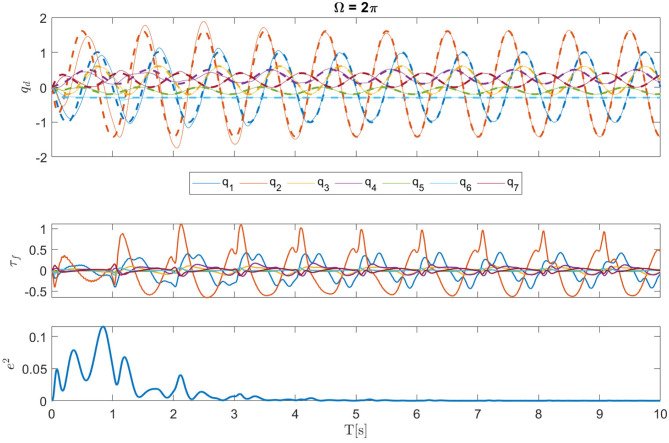
Top and middle plots show example joint and torque trajectories, respectively (Ω = 2π example). The bottom plot shows the sum of the square motion tracking error during the leaning process.

By using the proposed P-CMPs system we can see that the tracking error, and hence the learning of the internal dynamical model, is rapid and successful. In the left plot in Figure 5, we can see several examples of learning dynamics with a different frequency of motion. Despite the fact that the robot axes are not fully dynamically decoupled, the proposed P-CMPs system is able to successfully learn the internal dynamic models, i.e., corresponding feed-forward parameters, and thereby significantly reduce the tracking error. Hence learning was successful for all desired frequencies of motion. Note that the rate of learning does not depend on the frequency of movement, as can be seen on the right-hand side-plot in Figure 4.

The kinematic motion improvements and the evolution of the corresponding internal dynamical models, i.e., torque profiles, is for a Ω = 2π example shown in Figure 5. Here we can see from the bottom plot that tracking error is significantly reduced in a relatively short time. After about four periods of motion, the feed-forward torque signals converge to the final shape.

**Figure 6:**
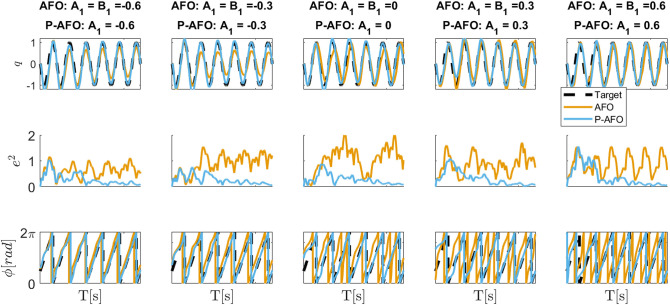
Difference between AFO and P-AFO system, both used with P-CMPs. The top plots show the desired and actual joint movements when using previously learned P-CMPs from the example in Figure 5. The middle plots show the tracking error and the bottom plots show the phase synchronization of CMPs.

In Figure 6 also we show why it is crucial to ensure that the phase ϕ is unambiguously defined considering the input signal. In this experiment we used the learned P-CMPs from the example in Figure 5 to compare the original AFO system with the P-AFO system. Note that both AFO and P-AFO systems were used with the proposed multi-layered control system based on P-CMPs. As expected when AFO is used, the feed-forward torque primitives might be shifted due to the properties of the original AFO approach. The phase shift of the torque primitives encoded in P-TPs, clearly results in a larger error compared to the new P-AFO approach which has an unambiguously define phase. As also shown in the example on Figure 3 the extracted phase of the P-AFO system is always clearly defined with respect to the input signal regardless of the initial conditions, while the extracted phase from the original AFO system might vary, with respect to the input signal. Note that an unambiguously defined phase is needed to ensure a reliable response of the P-CMPs.

### 4.3. Physical Interaction Example

**Figure 7:**
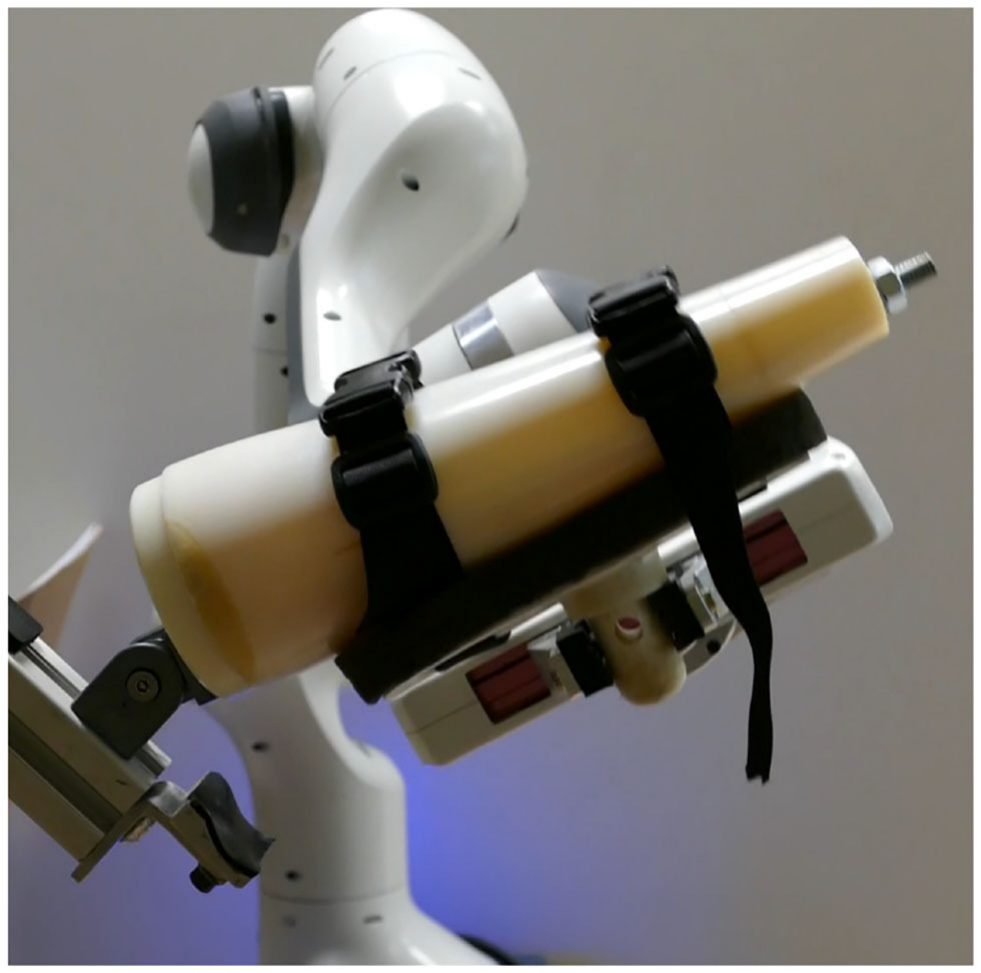
Experimental setup for physically simulated human elbow stretching tasks.

In the last example, the proposed P-CMPs method was demonstrated on a task where the robot was holding a human hand model with the simulated elbow joint as shown in Figure 7. In this experimental setup, we used a Franka Emika Panda robot. Note that the dimensional proportions and weight are equal to an adult human arm. The physical arm model is part of the evaluation of the possibility to help rehabilitation specialists during rehabilitation procedures. Here a typical strategy would be that the rehabilitation specialist defines the desired kinematic motion for rehabilitation using imitation learning. In such a case, considering that the robot could also hold an actual human hand, it would be difficult to obtain a mathematical dynamical model. Due to human variability, it would be a very specific, complex, and time-consuming task.

Instead, we can use the proposed P-CMPs approach to learn task-specific, appropriate torques for a given kinematic trajectory. This task could be performed with the original CMPs system combined with the statistical generalization. However, this would not be most effective since it would require to learn the CMPs at the specific frequency to build the database. In contrast, the proposed P-CMPs framework allows learning at an arbitrary frequency, as the frequency dependence is built into the P-TPs system. Working with a compliantly controlled robot, i.e., low feedback gains, with the ability to produce accurate trajectory tracking makes the system also safer for the environment, operator, and user.

**Figure 8:**
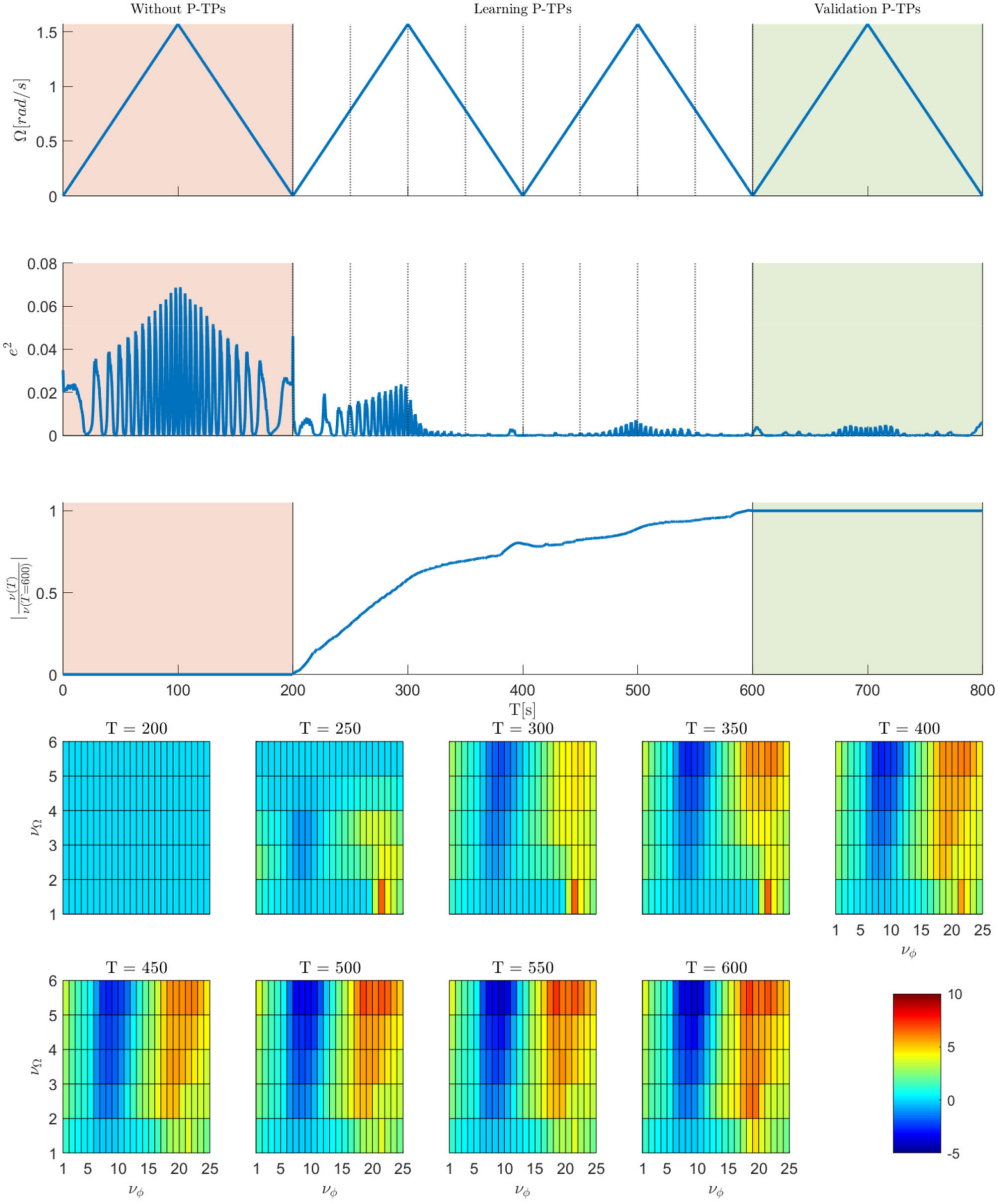
Results of elbow stretching example. The top plot shows the desired motion frequency. The second plot shows the sum of square tracking errors. The third plot shows the relationship between current and final weight matrix. Bottom plots show the P-TPs weight matrix values for one joint at a certain time during the learning process.

To show the P-CMPs performance, the kinematic motion for elbow stretching was defined by using kinesthetic teaching (Deniša et al., 2013). The robot feedback loop gains for all joints were set to 20 Nm/rad and the feed-back error learning parameter ι was set to 10. The performance of the P-CMPs framework for this example is shown in Figure 8, where we show in the top plot the desired frequency of motion, in the second plot the corresponding kinematic tracking error and in the third plot we show the relationship between current and final weight matrix for one joint. The experiment was divided into three parts, motion tracking without feed-forward P-TPs model, learning of P-TPs model, and validation of learned P-TPs model, respectively. The bottom plots show the evolution of P-TPs weights for one degree of freedom during the learning process. Note that P-TPs weights for one degree of freedom are a matrix **ν** with size of *M* × *K*, where *M* = 25 and *K* = 6. Note that *M* is the number of phase kernels, and *K* is the number of frequency kernels. Here the $c_{i}^{\phi }$, *i* = 1, 2, … *M* is equally distributed between 0 and 2π and $c_{j}^{\text{Ω}}$, *j* = 1, 2, … *K* is equally distributed between 0 and π.

The sum of square tracking errors shows that the proposed approach can significantly improve the kinematic tracking. We can see also that, by performing one sweep through the frequency space already significantly improves the tracking error. As seen in the third plot and bottom plots in Figure 8, at *T* = 400s the weights are already at the 80% of the final value. Note that in the second sweep through the frequency space the weights changes for less than 20% with respect to the weights value at *T* = 600s. Furthermore, the validation part shows that tracking error remains low, even after the learning process, as expected. Since the proposed P-CMPs approach remains parametric in terms of P-DMPs and P-TPs weights, all previously developed statistical methods can also be applied, allowing for further expansion of the task-specific learning of dynamics.

## 5. Conclusion

We presented a new P-CMPs framework consisting of a novel P-AFO frequency and phase synchronization systems, periodic DMPs, and a novel P-TPs system encoding task-specific primitives. The proposed P-CMPs system uses feedforward torque signals which are associated with corresponding kinematic motions. We showed, that the novel approach is able to unambiguously extract not only the frequency but also the phase from an arbitrary signal which allows anchoring the P-TPs to the P-DMPs trajectories. Furthermore, the novel extension of the P-TPs system also makes P-TPs frequency-dependent, which enables smooth frequency modulation of the P-CMPs. Integrating the feedback error learning concept in P-CMPs also improves the usability of the system. Our results indicate that the system was able to synchronize the kinematic and dynamics signals enabling compliant behavior while maintaining high tracking accuracy, without the need for developing mathematical dynamical models of the robot or the task.

The proposed P-CMPs framework is an improvement compared to the previews CMPs framework, enabling better learning performance and smooth frequency modulation abilities of periodic tasks.

## Data Availability Statement

The original contributions presented in the study are included in the article/supplementary material, further inquiries can be directed to the corresponding author/s.

## Author Contributions

TP contributed to the design, execution, and drafting of this work.

## Conflict of Interest

The author declares that the research was conducted in the absence of any commercial or financial relationships that could be construed as a potential conflict of interest.
